# Vestibule-Middle Ear Dehiscence Tested With Perilymph-Specific Protein Cochlin-Tomoprotein (CTP) Detection Test

**DOI:** 10.3389/fneur.2019.00047

**Published:** 2019-01-30

**Authors:** Takeshi Fujita, Takaaki Kobayashi, Kazuya Saito, Toru Seo, Tetsuo Ikezono, Katsumi Doi

**Affiliations:** ^1^Department of Otolaryngology, Kindai University Faculty of Medicine, Osaka, Japan; ^2^Department of Otolaryngology, Saitama Medical University, Saitama, Japan

**Keywords:** cochlin-tomoprotein, CTP, perilymph, fissula ante fenestram, otic capsule dehiscence, perilymphatic fistula, conductive hearing loss

## Abstract

An 8-year-old boy was referred to the ENT department for further evaluation of right-sided conductive hearing loss. A small cyst anterior to the oval window and fixation of the stapes footplate were observed during an exploratory tympanotomy. The concentration of a perilymph-specific protein, cochlin-tomoprotein (CTP), in the middle ear lavage fluid was measured with an ELISA-based CTP detection kit. The level of CTP in the middle ear lavage fluid before fenestration of the cyst was 0.26 ng/ml (negative), and its level after fenestration was 2.98 ng/ml (positive), confirming the presence of perilymph in the cyst. A small bone dehiscence, considered to be the fissula ante fenestram, was observed anterior to the stapes footplate after removal of the cyst. The CTP detection test results allowed us to confirm that the small bone dehiscence was connected to the inner ear.

## Introduction

After superior semicircular canal dehiscence syndrome (SSCD) was first described by Minor et al. (1), dehiscence has been found in various sites within the otic capsule, including posterior semicircular canal dehiscence, internal carotid artery-cochlea dehiscence, and posterior semicircular canal-jugular bulb dehiscence (2, 3). Recently, this clinical entity has been renamed as otic capsule dehiscence syndrome (OCDS) or third window syndrome (TWS) because these patients complain of similar symptoms and signs (2, 4).

The classic symptoms and signs of these syndromes are vertigo, nystagmus induced by loud noises (Tullio phenomenon) or increases in external auditory canal pressure (Hennebert sign), and a characteristic low-frequency air-bone gap with audiometry due to decreased air conduction and increased bone conduction (3, 5). Patients also present with numerous other symptoms such as hearing loss, vertigo, dizziness, nausea, pulsatile tinnitus, autophony, oscillopsia, headache, and cognitive dysfunction (2–4).

These symptoms and signs are not always present in the patients. Thus, surgery is important for making the definitive diagnosis when patients' symptoms and radiological findings suggest OCDS or TWS. However, it is usually hard to confirm that the dehiscence in the otic capsule is connected to the inner ear, even with surgery. In addition, the confirmation of existing perilymph in the middle ear is difficult. Even if fluid leakage is found in the middle ear, it is often difficult to distinguish perilymphatic flow from mucous membrane seepage, cerebrospinal fluid (CSF), or other fluids.

We treated an 8-year-old boy with right-sided conductive hearing loss caused by an open fissula ante fenestram (FAF) and fixed stapes footplate. During tympanotomy, we identified a cystic lesion anterior to the stapes. The fluid content of the lesion was identified as perilymph using a cochlin-tomoprotein (CTP) detection test. CTP, the shortest isoform of cochlin encoded by the *COCH* gene, has been proven to be a perilymph-specific protein and is used as a diagnostic biochemical marker for perilymph leakage (6–10). We present here the first case of vestibule-middle ear dehiscence with perilymph passage through the bony defect confirmed by biochemical assay.

## Case Report

**Figure 1:**
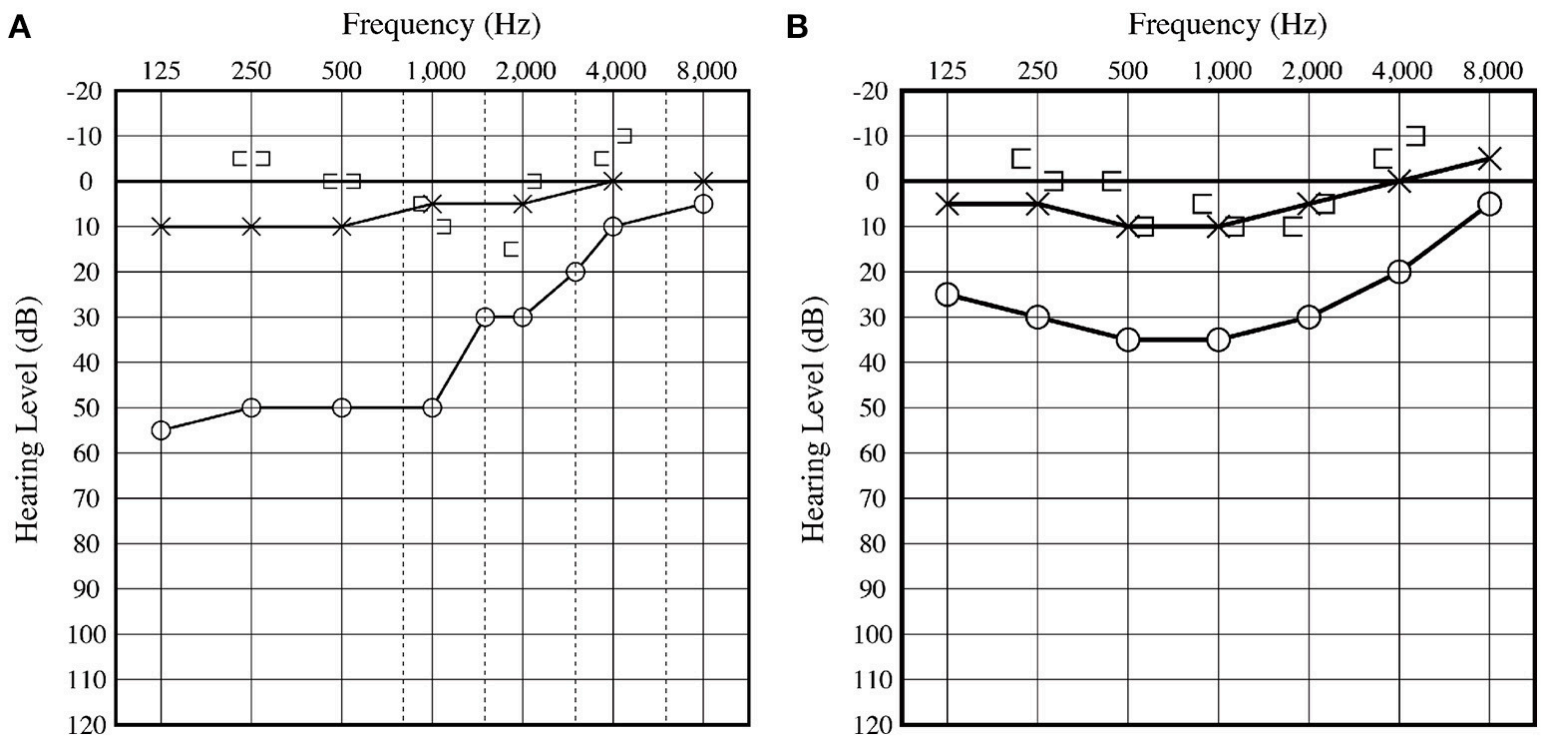
**(A)** Preoperative audiogram displays right-sided low frequency conductive hearing loss. **(B)** Postoperative audiogram displays slight hearing improvement at low frequency compared with the preoperative hearing level. Conductive hearing loss remained due to fixation of the footplate.

**Figure 2:**
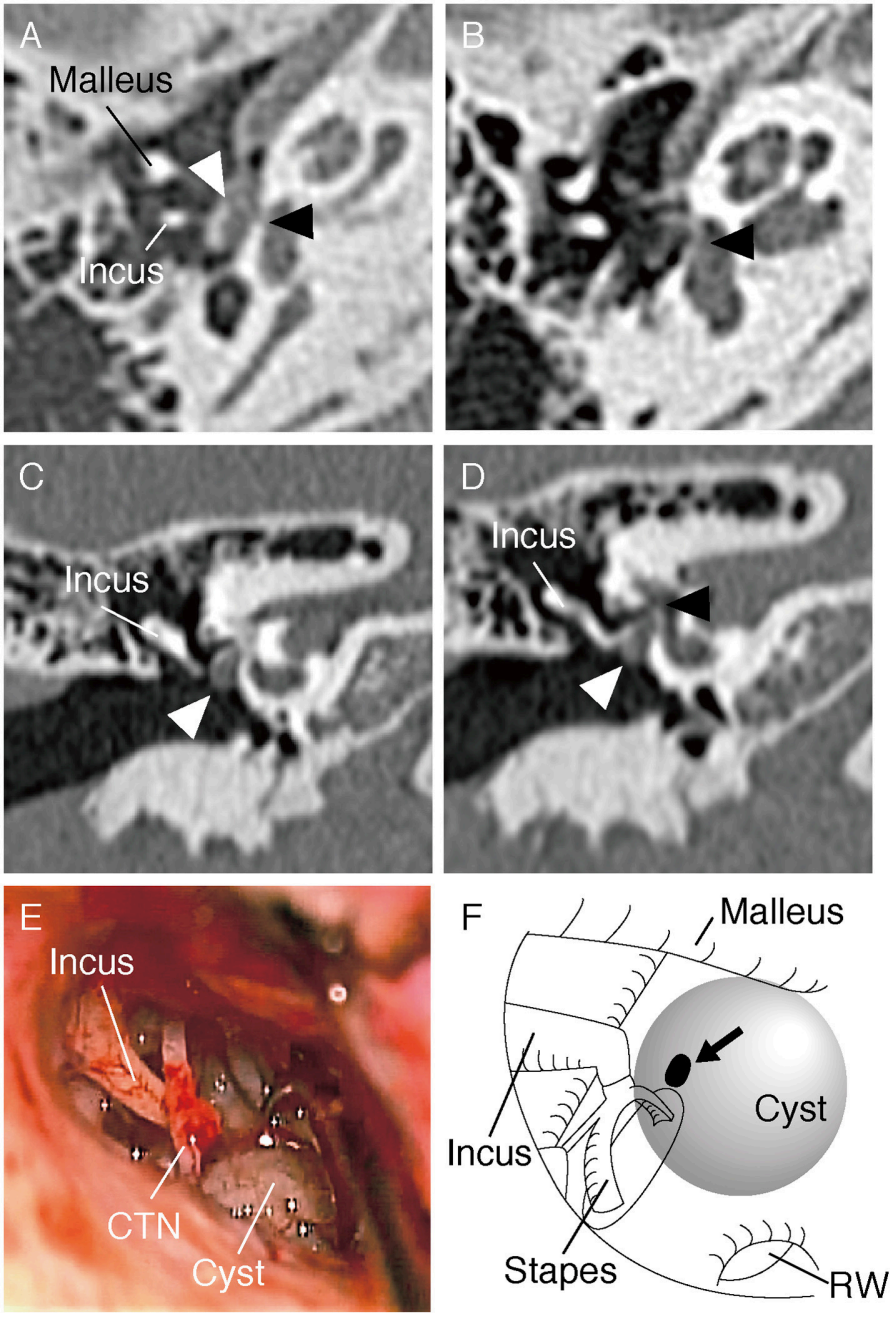
**(A,B)** Axial section of the CT scan of the right temporal bone. The cyst (white arrow head) and small bone dehiscence in the vestibule (black arrow head) are shown. **(C,D)** Coronal section of the CT scan shows the cyst (marked with a white arrow head) and small bone dehiscence in the vestibule (black arrow head). **(E)** Intraoperative picture during exploratory tympanotomy. A cyst can be seen anteroinferior to the oval window. CTN, Chorda tympani nerve. **(F)** Schematic illustration of the middle ear in the surgery. A small bone dehiscence (marked with an arrow) was found anterior to the stapes footplate after removal of the cyst. RW, round window.

An 8-year-old boy was referred to our hospital for further evaluation of right-sided conductive hearing loss identified at a health check-up at school (Figure 1A). He did not show any other symptoms related to third window syndrome, such as sound-induced dizziness, nausea, autophony or headache (2). Serial computed tomography (CT) imaging showed a small soft-tissue density lesion close to the oval window (Figures 2A,C,D). A small bone dehiscence within the otic capsule was also indicated in the CT images (Figures 2A,B,D). A small cyst (anterior to the oval window) and fixation of the stapes footplate were found during an exploratory tympanotomy (Figure 2E). To investigate the nature of the content fluid of the cyst, we fenestrated the cyst wall. Middle ear lavage fluid (MEL) was taken before and after the opening procedure. CTP concentration in the MEL before fenestration was 0.26 ng/ml (negative), and after fenestration was 2.98 ng/ml (positive), which confirmed the presence of perilymph in the cyst. A small bone dehiscence, considered to be a FAF, was found anterior to the stapes footplate after removal of the cyst (Figure 2F). The small bone dehiscence was sealed with connective tissue and fibrin glue. In the postoperative audiogram, conductive hearing loss improved by 15–20 dB at a low frequency but was still present due to fixation of the footplate (Figure 1B). The conductive hearing loss in this case was caused not only by the cyst but also by another middle ear anomaly: stapes footplate fixation. We plan to perform stapes surgery as the second-stage surgery.

## CTP Measurement

Details of the CTP detection test method have been previously described (10, 11). In brief, middle ear lavage fluid samples (MEL) for the test were taken as follows: (1) the middle ear was washed 3 times with 0.3 ml saline, (2) the fluid was recovered, and red blood cells and white blood cells were removed from MEL by centrifugation (2,000 g, 3 min), and (3) the supernatant was then collected and frozen. We defined the diagnostic cutoff criteria as follows: CTP < 0.4 = negative; 0.4 ≤ CTP < 0.8 = intermediate; and 0.8 ≤ CTP (ng/ml) = positive. CTP was measured by one of the authors (T.I.) in a multicenter investigator-initiated clinical trial using a novel ELISA-based CTP detection kit from SRL, Inc. (SRL Inc., Tokyo, Japan) (10, 11). The details of the ELISA-based CTP detection are as follows: An Immuno Module Plate (Nalge Nunc, Rochester, NY, USA) was coated with a mixture of anti-CTP (in 0.1 mol/L carbonate buffer, pH 9.5) and incubated at 4°C overnight, and then blocked with 1% bovine serum albumin in phosphate buffered saline (PBS). The samples and standard recombinant human CTP (rhCTP) proteins were diluted to 10-fold with a dilution buffer (0.05% Tween 20 in PBS) and 100 μL of samples was added to each well, followed by incubation of the samples at 37°C for 1 h. After nine washes with a washing buffer, 100 μL of horseradish peroxidase-labeled anti-CTP antibody was added to each well, followed by incubation at 4°C for 30 min. After nine washes with the washing buffer, 100 μL of tetramethyl benzidine buffer was added as a substrate to each well, followed by incubation in the dark at room temperature for 30 min. Color development was stopped by the addition of 100 μL of the stop solution (1N H_2_SO_4_). The optical density of each sample at 450 nm was then measured. Samples were measured in duplicate, and serially diluted rhCTP (*E. coli*) was used to generate a standard curve. The concentration of CTP was calculated from the standard curve by using the linear regression method. It takes approximately 2 weeks to obtain the test results (10).

## Discussion

Fissula ante fenestram (FAF) is a slit-like opening anterior to the oval window, an anatomical variation of the bony labyrinth (12). A FAF may be observed in each individual from birth until early childhood, with the bony cleft filled with hyaline cartilage and mesenchyme. During adolescence, the FAF gradually disappears due to bone remodeling of the otic capsule (12–14). In this case study, altered remodeling possibly resulted in remnant soft tissue on the bony cleft, and this may have caused cyst formation via perilymph flow through the FAF. In the present case, HRCT showed a possible bone defect in the anterior part of the footplate, however, we identified a dehiscence not on the footplate but at the site of the FAF.

Conductive hearing loss implies a mechanical problem in the outer or middle ear. In this case, the patient's outer ear and tympanic membrane were normal. His conductive hearing loss was considered to be due to ossicular malformation or other middle ear malformations. To confirm the diagnosis, we performed an exploratory tympanotomy. The improvement of conductive hearing loss by 15–20 dB at a low frequency (Figure 1) was considered to be a result of sealing the otic capsule dehiscence. A third window in the inner ear causes a low-frequency air-bone gap due to the dual mechanism of worsening air conduction thresholds and improvement of bone conduction (5). The air-bone gap resulting from the third window boosts the conductive hearing loss resulting from the stapes fixation.

To date, the phenotype of TWS has been reported in association with superior semicircular canal dehiscence (SSCD), posterior canal dehiscence, posterior canal-jugular bulb dehiscence, cochlea-carotid dehiscence, cochlea-facial nerve dehiscence, and wide vestibular aqueduct (2). Nevertheless, in a previous study, the typical symptoms of these TWSs, such as vertigo and autophony, were observed in less than half of the patients with TWS (3). Lack of the typical symptoms does not exclude the existence of the third window in the inner ear. The definitive diagnosis of TWS is necessary to be established during the surgery. All of these, with the exception of wide vestibular aqueduct, are possible perilymph fistulae. The FAF is considered a potential pathway for perilymph leakage (15, 16). However, even if fluid leakage is observed in the FAF region intraoperatively, it is usually difficult to distinguish perilymphatic flow from mucous membrane seepage, cerebrospinal fluid (CSF), or other fluids.

CTP, a 16-kDa short isoform of cochlin, has been identified only in the perilymph (17). Cochlin is the major component, after collagen, of the extracellular matrix in the inner ear and is considered to be involved in immune responses in the inner ear (18). Although the precise role of CTP in the inner ear remains unknown, its usefulness as a marker of perilymph has been reported in several cases (11, 19–21). The sensitivity and specificity of CTP as a means of diagnosing perilymph fistula in cochlear implantation surgery have been reported to be 92.3and 98.2%, respectively, (8, 9). The CTP detection test results allowed us to confirm that the small bone dehiscence in this case was connected to the perilymphatic space of the vestibule.

The CTP detection test is helpful in evaluating the pathophysiology of hearing loss or vertigo by verifying the existence of perilymph. There are some reports that CT-negative third window syndromes with exactly the same clinical phenotype also exist (2). Those cases are also possible perilymph fistulae. The CTP detection test could be a remarkable diagnostic tool for such difficult and controversial cases.

## Conclusion

We report the first case of an otic capsule dehiscence confirmed using a CTP ELISA detection test. The CTP detection test is extremely useful to verify the existence of perilymph in the middle ear.

## Ethics Statement

Written informed consent was obtained from the legal guardians of the participant for the publication of this case report.

## Author Contributions

TF, TK, KS, and KD reviewed the literature and collected data. TF made figures and wrote a first version of the manuscript. TI, TS, and KD edited the manuscript. All authors contributed to the final version of the manuscript.

### Conflict of Interest Statement

Saitama Medical University has the patents for the hCTP ELISA test. The development of this hCTP ELISA was performed in collaboration with IBL Inc. under license from (and with) the technical assistance of Saitama Medical University with royalties. The authors declare that the research was conducted in the absence of any commercial or financial relationships that could be construed as a potential conflict of interest.
